# TDP-43 Amyloid Fibril Formation via Phase Separation-Related and -Unrelated Pathways

**DOI:** 10.1021/acschemneuro.4c00503

**Published:** 2024-10-03

**Authors:** Pin-Han Lin, Guan-Wei Wu, Yu-Hao Lin, Jing-Rou Huang, U-Ser Jeng, Wei-Min Liu, Jie-rong Huang

**Affiliations:** † Institute of Biochemistry and Molecular Biology, National Yang Ming Chiao Tung University, No. 155 Section 2, Li-nong Street, Taipei 11221, Taiwan; ‡ Institute of Biomedical Informatics, National Yang Ming Chiao Tung University, No. 155 Section 2, Li-nong Street, Taipei 11221, Taiwan; § Department of Life Sciences and Institute of Genome Sciences, National Yang Ming Chiao Tung University, No. 155 Section 2, Li-nong Street, Taipei 11221, Taiwan; ∥ National Synchrotron Radiation Research Center, Hsinchu 30076, Taiwan; ⊥ Department of Chemistry, Fu Jen Catholic University, No.510, Zhongzheng Rd., New Taipei City 24205, Taiwan

**Keywords:** TDP-43, liquid−liquid phase separation, amyloid fibril, intrinsically disordered proteins, NMR, wide-angle X-ray scattering

## Abstract

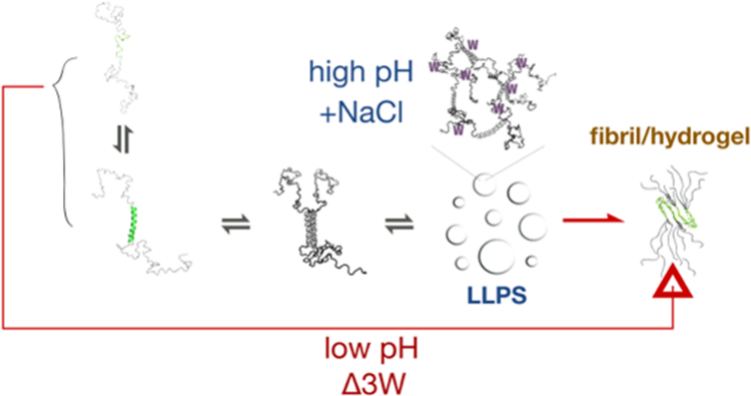

Intrinsically disordered regions (IDRs) in proteins can undergo liquid–liquid phase separation (LLPS) for functional assembly, but this increases the chance of forming disease-associated amyloid fibrils. Not all amyloid fibrils form through LLPS however, and the importance of LLPS relative to other pathways in fibril formation remains unclear. We investigated this question in TDP-43, a motor neuron disease and dementia-causing protein that undergoes LLPS, using thioflavin T (ThT) fluorescence, NMR, transmission electron microscopy (TEM), and wide-angle X-ray scattering (WAXS) experiments. Using a fluorescence probe modified from ThT strategically designed for targeting protein assembly rather than β-sheets and supported by TEM images, we propose that the biphasic ThT signals observed under LLPS-favoring conditions are due to the presence of amorphous aggregates. These aggregates represent an intermediate state that diverges from the direct pathway to β-sheet-dominant fibrils. Under non-LLPS conditions in contrast (at low pH or at physiological conditions in a construct with key LLPS residues removed), the protein forms a hydrogel. Real-time WAXS data, ThT signals, and TEM images collectively demonstrate that the gelation process circumvents LLPS and yet still results in the formation of fibril-like structural networks. We suggest that the IDR of TDP-43 forms disease-causing amyloid fibrils regardless of the formation pathway. Our findings shed light on why both LLPS-promoting and LLPS-inhibiting mutants are found in TDP-43-related diseases.

## Introduction

Over half of human proteins are intrinsically disordered or contain intrinsically disordered regions (IDRs).^1^ Many IDRs have prion-like characteristics and are implicated in neurodegenerative diseases, including the extensively studied hnRNP A1, FUS, and TIA1.^2^ The high prevalence of disease-associated IDRs in the proteome was a long-standing enigma until recent findings revealed their ability to undergo liquid–liquid phase separation (LLPS).^3^ This physicochemical process underlies the spatiotemporal modulation of many cellular functions^4,5^ but comes at the cost of an increased tendency for pathological fibril formation.^6,7^ This paradigm-shifting phenomenon has subsequently been found to occur in several proteins associated with neurodegenerative diseases, including α-synuclein,^8^ amyloid-β,^9,10^ and tau protein.^9,11,12^ Recent studies of these proteins have mostly found that amyloid fibril formation is promoted by LLPS,^8,13,14^ but non-LLPS-driven fibrilization has also been reported,^15^ and the relative importance of LLPS and other pathways in fibril formation remains to be elucidated.

**Figure 1:**
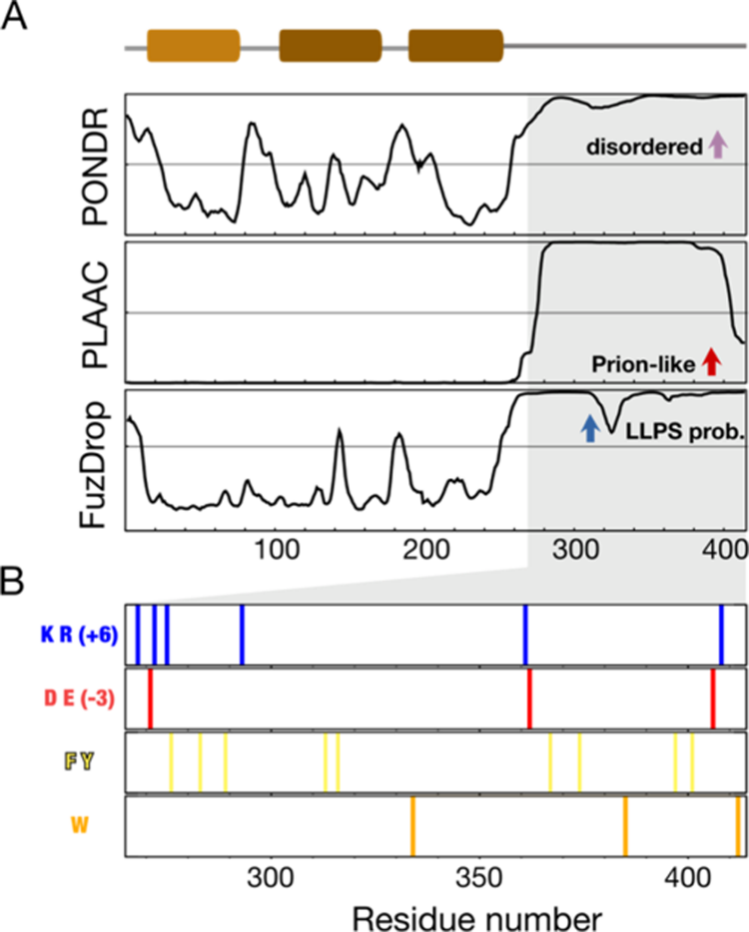
TDP-43 sequence analysis. (A) Upper panel: structured regions including the N-terminal domain (light brown) and two RNA-recognition motifs (brown). Lower panel: levels of structural disorder (PONDR), prion-likeness (PLAAC), and tendency to undergo liquid–liquid phase separation (FuzDrop) predicted from the primary sequence (UniProt entry: Q13148). (B) Specific types of amino acids labeled along the amino-acid sequence of the intrinsically disordered region. K/R: lysine/arginine; D/E: aspartate/glutamate; F/Y: phenylalanine/tyrosine; W: tryptophan.

Transactive response DNA-binding protein of 43 kDa (TDP-43) is implicated in a variety of motor neuron diseases and dementia.^16^ TDP-43 consists of a structured N-terminal domain that promotes dimerization, a pair of RNA-recognition motifs, and an IDR spanning residues ∼260–414 (Figure 1A).^17^ The transient formation of an α-helix in the IDR from residues 320 to 340 is critical for self-assembly,^18−20^ predominantly driven by hydrophobic interactions.^21^ This α-helical region has also been shown to transform into β-sheets that assemble into amyloid fibrils.^22−25^ TDP-43 is also an extensively studied model for understanding protein LLPS. The self-assembly process is not only tuned by the transient α-helix in the IDR^26^ but also by aromatic residues, which act as “stickers”, facilitating multivalent cross-linking.^27^ The three tryptophans in the IDR (Figure 1B) and the α-helix are evolutionarily conserved, and date back to prevertebrate species.^28^ The assembly process is also influenced by various other factors, including the presence of ions,^29^ small molecules,^30^ and other biomolecules.^31−33^ The phase-separated state of TDP-43 is known to be an amyloid intermediate,^24,34^ but the 50-or-so known disease-associated TDP-43 mutations are not all LLPS-promoting.^17,35^ For example, the A315E mutation, which mimics the phosphorylated state of A315T, tends to promote LLPS, but the Q331K mutation hinders LLPS.^20,21,27^ These contrasting behaviors make TDP-43 an interesting model in which to investigate the balance between LLPS and non-LLPS pathways to fibrilization in neurodegenerative diseases.

## Results and Discussion

### TDP-43 Fibrilizes Faster at LLPS-Promoting Conditions

**Figure 2:**
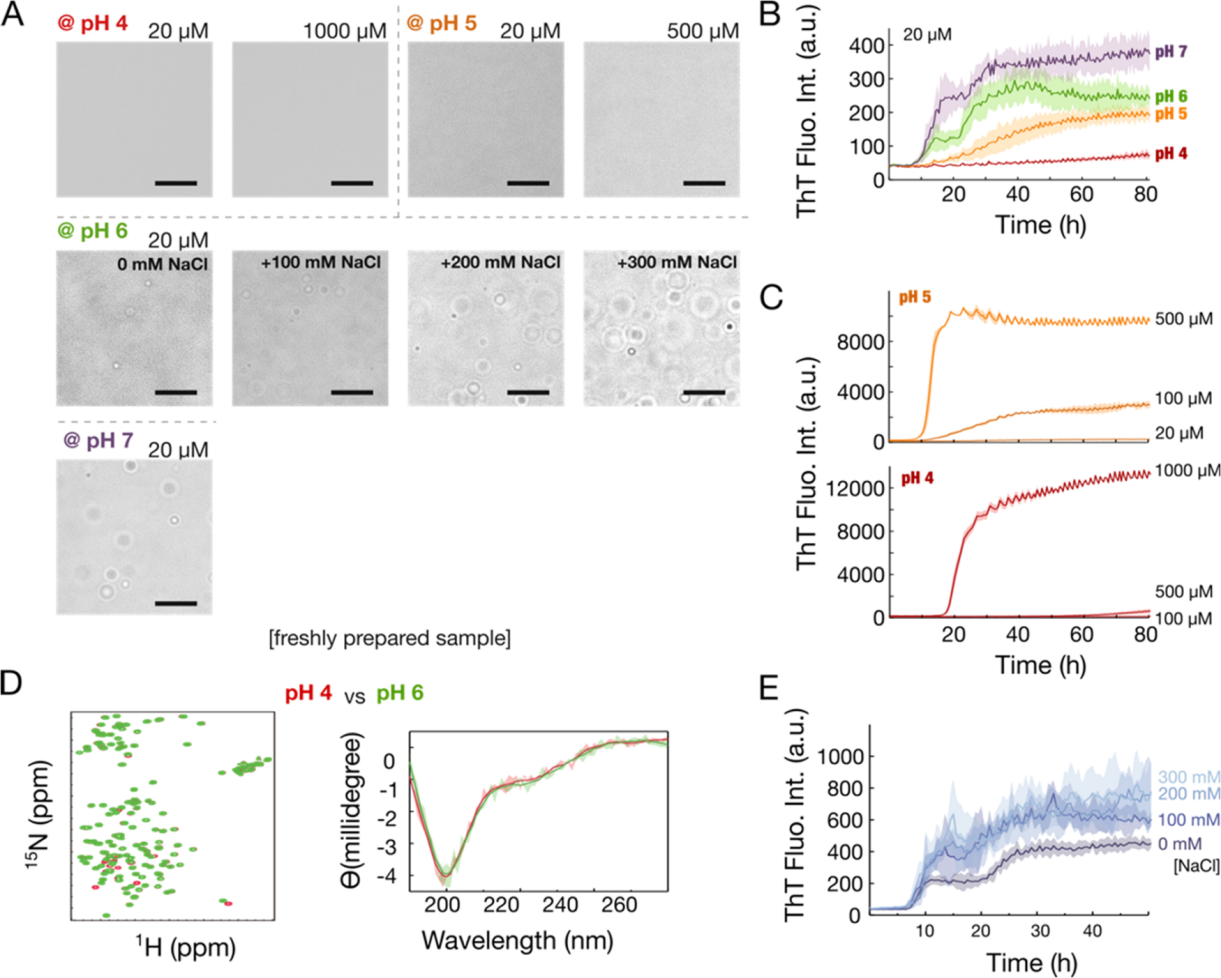
Phase separation and thioflavin T (ThT) fluorescence analysis of TDP-43 at various conditions. (A) Microscopic images of different TDP-43 concentrations at pH 4–7. At pH 6, 0–300 mM NaCl was added. Scale bar: 10 μm. Samples were freshly prepared. (B) ThT assays at pH 4–7 with 20 μM protein samples. (C) ThT assays at pH 5 and 4 at high protein concentrations. (D) Overlaid ^1^H–^15^N HSQC and CD spectra of proteins at pH 4 (red) and pH 6 (green) of freshly prepared samples, indicating similar structure propensities. (E) ThT assays of 20 μM protein samples at NaCl concentrations of 0, 100, 200, and 300 mM.

TDP-43’s IDR has a net charge of +3 (Figure 1B). Low pH, therefore, reduces its propensity to undergo LLPS because it increases the protonation of charged residues, leading to net electrostatic repulsion. Also, the presence of NaCl screens out electrostatic repulsion, as demonstrated by our group^21^ and others,^34^ thereby lowering the phase separation threshold. As expected, no condensates were observed at pH 4, even at a concentration of 1 mM (Figure 2A). Conversely, at pH above 6, protein condensation occurs more readily, and this effect is further enhanced in the presence of NaCl (Figure 2A). Correspondently, thioflavin T (ThT) assays, which are commonly used to detect amyloid fibrils, indicated slower fibril formation kinetics (a slower increase in ThT fluorescence) at lower pH (4 or 5) than at pH 6 or 7 with the same protein concentrations (Figure 2B). This slowdown of fibril formation at low pH is offset by increasing the protein concentration (Figure 2C). The slower rate of fibril formation is independent of conformation change (i.e., the denature of the middle α-helical structure) because NMR and CD spectra of freshly prepared samples at pH 4 and 6 (Figure 2D) indicate similar secondary structure propensities. Similarly, adding NaCl, thereby mitigating electrostatic effects and shifting the equilibrium toward assembled states, led to increased ThT fluorescence (Figure 2E), indicating faster kinetics of fibril formation correlates to the conditions that favor LLPS of TDP-43. However, regardless of whether TDP-43 undergoes LLPS, it forms amyloid fibril. We next thus investigated potential mechanisms behind the phase separation-related and unrelated pathways.

### Biphasic ThT Kinetics Correlates to the Presence of Amorphous Assembly

**Figure 3:**
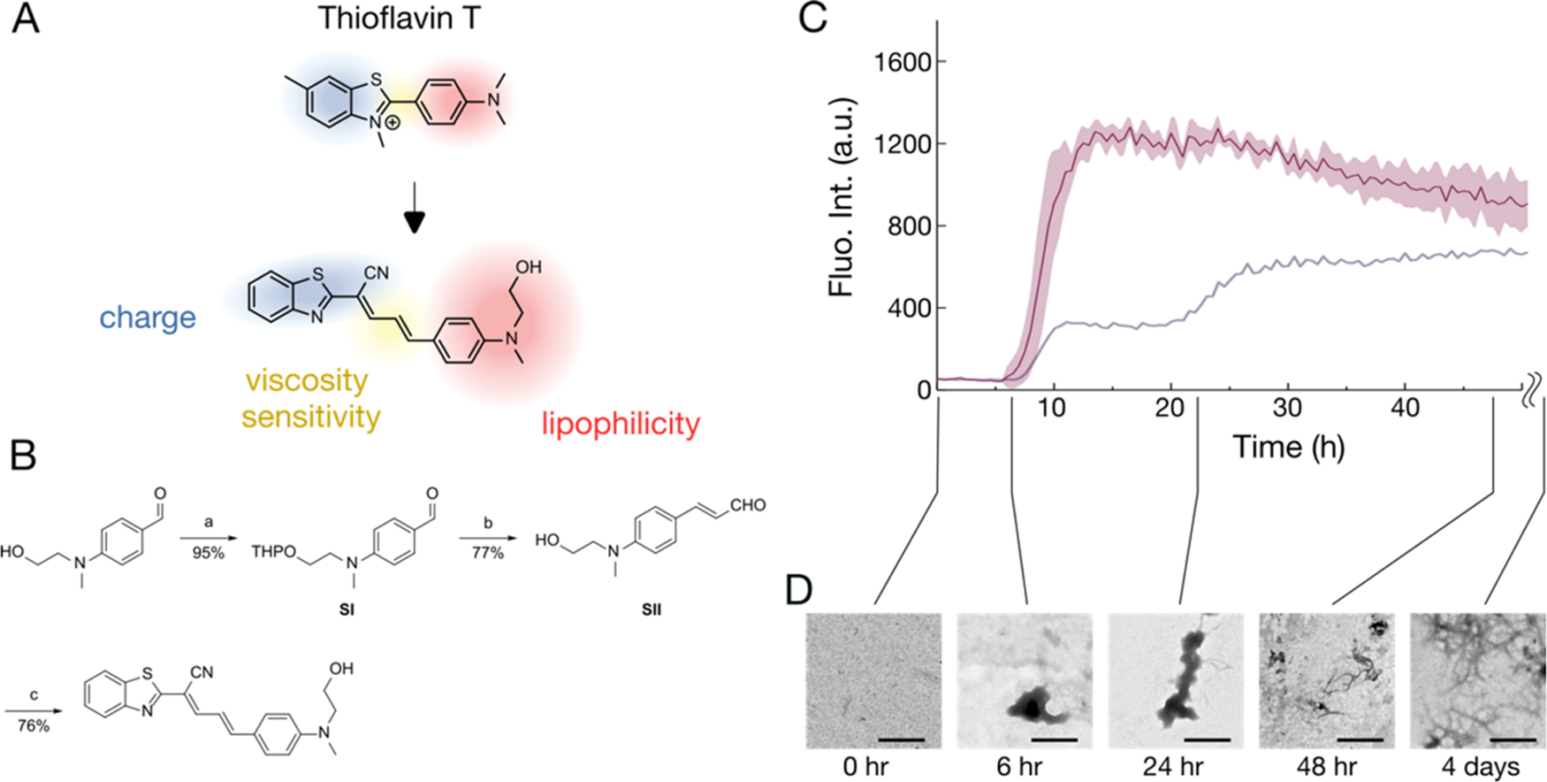
Schematic illustration of the chemical structure and fluorescence of the modified probe (ThT^ene–OH^). (A) Chemical structures of ThT^ene–OH^ and of thioflavin T (ThT), with the rationale for the modifications in colored text and shading. (B) Synthesis of ThT^ene–OH^. Reagents and reaction conditions: (a) 3,4-dihydro-*2H*-pyran, pyridinium *p*-toluenesulfonate, CH_2_Cl_2_, rt, 37 h; (b) (i) (1,3-dioxolan-2-ylmethyl)triphenylphosphonium bromide, 60% NaH, THF, reflux, 6.5 h; (ii) 2 M HCl _(aq.)_, THF, rt, 20 h; (c) 2-benzothiazoleacetonitrile, piperidine, EtOH, reflux, 14.5 h. (C) Fluorescence signal from ThT^ene–OH^ for a 20 μM protein sample at pH 6 in red, with the ThT signal under the same condition in gray. (D) The transmission electron microscopic images of the samples incubated for the indicated periods. Scale bar: 500 nm.

Under the condition that phase separation was observed (Figure 2A), i.e., at pH 6 and above or in the presence of NaCl, the fluorescence increase in ThT assays occurred in two stages (Figure 2B,E). Although ThT is widely used to monitor fibrillization, as a charged molecule, its binding affinity is pH- and ion-concentration-dependent.^36^ Also, protein aggregation typically begins with forming low-viscosity soluble protein oligomers before high-viscosity insoluble protein aggregates gradually form.^37,38^ Therefore, to make the assays more sensitive to the properties of the intermediate phase and to reduce the risk of unwanted electrostatic interactions,^39^ we strategically designed a probe based on the structure of ThT. The methylbenzothiazole group in ThT was replaced with a 2-benzothiazoleacetonitrile moiety to eliminate the positive charge (blue shading in Figure 3A). The π conjugation was extended to improve the viscosity sensitivity toward the environment, enabling fluorescence at lower viscosities (yellow in Figure 3A).^37,38^ One of the two methyl groups attached to the nitrogen atom fragment was replaced by an ethyl alcohol group to adjust the lipophilicity (red in Figure 3A).^40^ We denoted this probe as ThT^ene–OH^ for this specific task. The synthetic scheme is shown in Figure 3B. Further details on the synthesis and characterization of this probe are given in the Supporting Information.

In our fluorescence assays with TDP-43, the increase in fluorescence intensity using ThT^ene–OH^ coincided with the first step observed with ThT (Figure 3C). Since ThT^ene–OH^ targets aggregates, irrespective of their β-sheet structure, we infer that the first step in the ThT assays indicates the presence of a condensed form of TDP-43 under conditions that promote self-assembly. Additionally, negative-stain transmission electron microscopy (TEM) images collected at synchronized time points of ThT assays show amorphous aggregates within 1 day, preceding the extensive amyloid fibril network formation (Figure 3D).

The biphasic kinetics observed in our assays, combined with TEM results, indicate that TDP-43 may form amorphous assemblies as an alternative pathway before transitioning to β-sheet-dominant amyloid fibrils. This behavior is consistent with observations in other amyloid systems, such as amyloid-β and α-synuclein, where oligomeric intermediates serve as precursors to mature fibrils.^41,42^ More recently, the concepts of microphase separation^43^ or nanocondensates^44^ from polymer chemistry have also been applied to protein assembly.^45^ The amorphous aggregates observed here via TEM might also represent similar metastable states preceding the formation of large condensates or amyloid fibrils. Characterizing these early assemblies of TDP-43 is beyond the current scope of our study but remains a promising avenue for future research to further elucidate the mechanisms of TDP-43 amyloid formation. Nevertheless, our findings, supported by strategically designed probes and TEM evidence, indicate that TDP-43 forms amorphous aggregates under conditions promoting LLPS before extensive amyloid fibril formation.

### TDP-43 Hydrogel Formation and Structural Characteristics at Low pH

**Figure 4:**
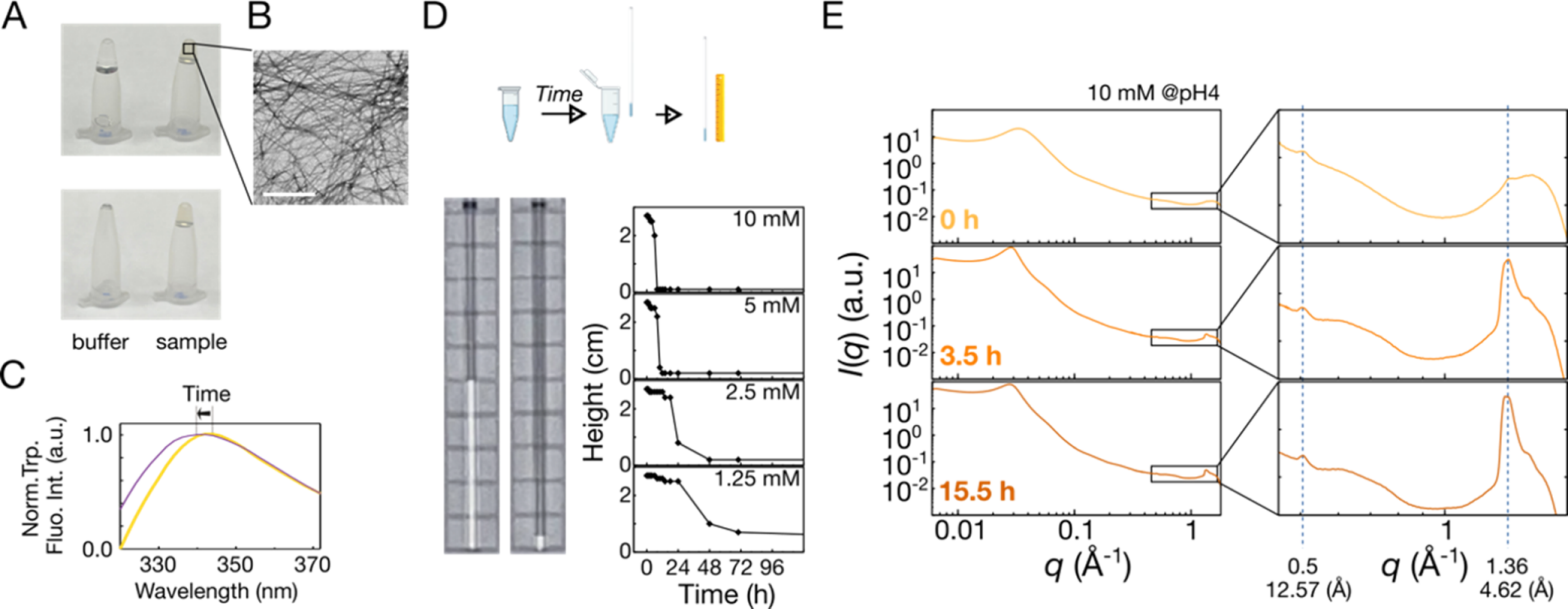
Gelation of TDP-43 at pH 4. (A) Representative photographs of gel and buffer samples, highlighting the greater viscosity of the former. The images depict the buffer (left) and protein sample (right; 10 mM at pH 4 incubated for days at room temperature) in microcentrifuge tubes immediately after inversion (top panel) and a few moments later with gentle tapping (bottom panel). The protein sample did not flow down, indicating gel formation and lack of fluidity. (B) The transmission electron microscopic image of the 1 mM sample incubated at 30 °C after 1 week. Scale bar: 500 nm. (C) Tryptophan fluorescence of samples in soluble (orange) and gel (purple) states. (D) Schematic illustration of gelation monitoring using capillary tubes and results obtained for different sample concentrations (1.25–10 mM). (E) Small/wide-angle X-ray scattering data at various incubation times. The highlighted regions correspond to the hydrogen bonding distance between β-strands and the intersheet distances in fibrils.

At low pH, ThT fluorescence increased in a single step, and no LLPS or amorphous aggregates were observed (Figure 2). When the sample concentration was increased to the millimolar range at pH 4, the sample became hydrogel within days (Figure 4A). A typical amyloid fibril morphology was observed from the TEM of the hydrogel (Figure 4B). This transition was accompanied by a blue shift in tryptophan fluorescence peak intensity, indicating that aromatic residues became encapsulated during the formation of the hydrogel (Figure 4C). The gelation kinetics were tracked using capillary tubes. Gelation was completed within 24 h at concentrations above 5 mM (Figure 4D), and the gelation process was also monitored in real time under these conditions using wide-angle X-ray scattering (WAXS; Figure 4E). Diffraction signals corresponding to approximately 4.6 and 12 Å were observed, typical of hydrogen bonding between β-strands and intersheet distances in amyloid fibrils.^15,24,46−48^ The β-strands may arise from the transition of α-helix in the IDR to a β-sheet arrangement, revealed in solution NMR spectrum by a decrease in peak intensity in the α-helical region.^49^ Therefore, the increased ThT signals, TEM images, and WAXS patterns indicate that the gel form of TDP-43 keeps the characteristics of amyloid fibrils.

### TDP-43 LLPS-Deprived Mutant Forms a Hydrogel under Neutral pH

**Figure 5:**
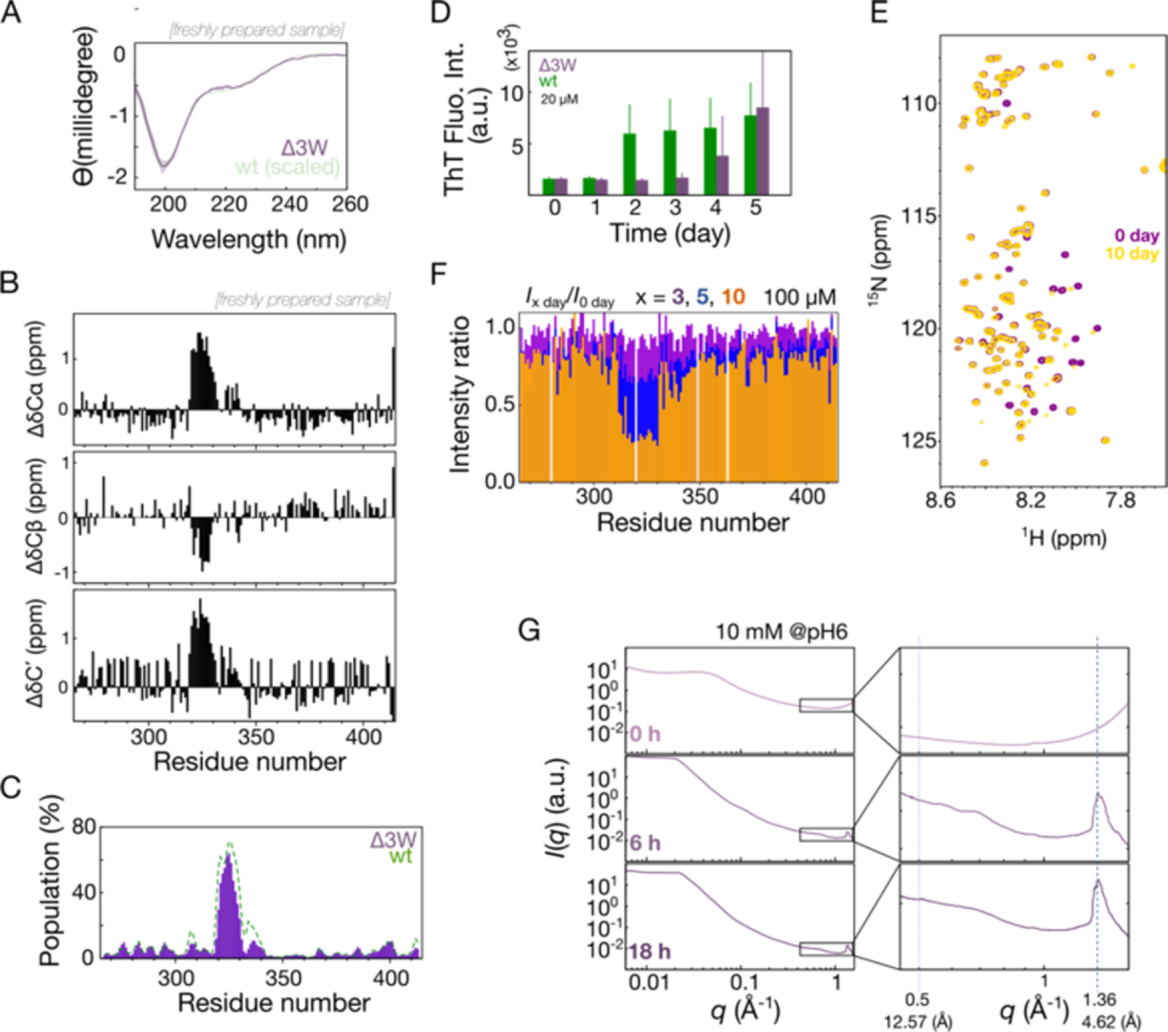
Structure and gelation analysis of a Δ3W (W334G/W385G/W412G) mutant of TDP-43. (A) CD spectrum of 20 μM Δ3W (purple), with the scaled wild-type spectrum in the background. (B) Δ3W secondary chemical shifts. (C) δ2D-estimated α-helix populations of the Δ3W mutant (purple) and wild-type TDP-43 (green). (D) Thioflavin T fluorescence from 20 μM wild-type (green) and Δ3W (purple) samples as a function of incubation time. (E) Overlaid ^1^H–^15^N HSQC spectra of freshly prepared Δ3W (purple) and of Δ3W after 10 days’ incubation (orange). (F) Intensity ratios of HSQC cross-peaks from samples incubated for 3, 5, and 10 days relative to those measured in a freshly prepared sample. (G) Small/wide-angle X-ray scattering data at various incubation times. The highlighted regions correspond to the hydrogen bonding distance between β-strands and the intersheet distances in fibrils.

Although TDP-43 can experience acidic environments inside cells, such as in lysosomes or in stress granules induced by intensive metabolic activity,^50,51^ we also examined the gelation properties at more neutral pHs in a construct (Δ3W) with reduced LLPS propensity. (LLPS in TDP-43 is nearly abolished when three key tryptophans are replaced with glycine, W334G/W385G/W412G).^27^ The CD spectra of Δ3W are similar to those of wild-type TDP-43 (Figure 5A). Moreover, we assigned the NMR chemical shifts of Δ3W (deposited in BMRB: 52369), and secondary chemical shifts analysis indicates that the α-helical element around residues 320–340 is still present (Figure 5B). The secondary structure composition predicted by δ2D from these shifts was similar to the wild-type for the α-helix, confirming that these mutations have little effect on the secondary structure (Figure 5C). In ThT assays nevertheless, the Δ3W construct formed amyloid fibrils more slowly than the wild type did (Figure 5D). The corresponding decrease in ^1^H–^15^N HSQC peaks from the α-helical region (Figure 5E,F) indicates that this region was involved in the transition. The WAXS monitoring of the gelation process revealed a weaker signal for the intersheet distance (∼12 Å) of the Δ3W fibrils compared to the wild-type (Figure 5E). This suggests a more heterogeneous cross-linking in the Δ3W fibrils within the gel form. Nevertheless, the diffraction signals corresponding to inter-β-strand hydrogen bonding (4.6 Å) remained detectable, similar to the wild-type hydrogel.

Both the wild-type sample at pH 4 and the LLPS-deprived construct at physiological pH exhibit ThT signals, characteristic inter-β-strand patterns from WAXS, and comparable fibril morphologies from TEM, suggesting that the hydrogel preserves the fibril structure. Although some hydrogels, such as those formed by IDRs of FUS^15,52,53^ or hnRNP A1,^54^ show reversible disassembly upon changes in temperature, concentration, or addition of denaturants, our hydrogel samples do not reversibly disassemble regardless of temperature changes, dilutions, or under strongly denaturing conditions (8 M urea, pH 2.5). Collectively, the TDP-43 hydrogel maintains a similar cross-β structure to that of amyloid fibrils.

## Conclusions

**Figure 6:**
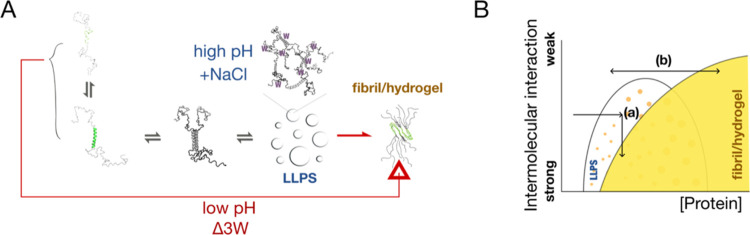
Schematic representation of the conclusions in the study. (A) LLPS and non-LLPS pathways for TDP-43 amyloid fibril formation. (B) Proposed multiphase diagram of TDP-43 under different conditions: Proteins under conditions (a) enter a reversible demixing state and can transition to the fibril state; proteins under conditions (b) bypass the reversible demixing state and directly enter the fibril/hydrogel state at high concentrations.

Although recent studies have highlighted the LLPS pathway toward amyloid formation,^8,13,14^ the present study, using TDP-43’s IDR as a model, demonstrates that amyloid fibril formation can occur without LLPS (Figure 6A). These findings can be coherently integrated into a multistate phase diagram (Figure 6B).^5^ Under conditions that promote intermolecular interactions (e.g., pH above 6 or the presence of salt), proteins undergo reversible demixing. As these intermolecular interactions intensify (for instance, due to prolonged incubation), the proteins transition into a dynamically arrested amyloid state (line (a) in Figure 6B). Conversely, when interprotein interactions are weak (such as in TDP-43 at pH 4 and the Δ3W construct), proteins only fibrilize or gelate when highly concentrated (line (b)). The two pathways in this model would explain why LLPS-promoting and LLPS-inhibiting mutants in TDP-43’s IDR can both be pathological and provide a mechanistic basis for TDP-43-related diseases.

## Materials and Methods

### Preparation of ThT^ene–OH^

The precursor of ThT^ene–OH^ was prepared according to the reported literature, and the details are shown in the Supporting Information. To a solution of 2-benzothiazoleacetonitrile (39 mg, 0.22 mmol) and compound **SII** (46 mg, 0.22 mmol) in absolute ethanol (5 mL) was added three drops of piperidine. The resulting mixture was heated to reflux for 14.5 h. The reaction mixture was then cooled to room temperature and the solvent was removed under reduced pressure. The concentrated crude residue was recrystallized from CH_2_Cl_2_/MeOH cosolvent to give 61 mg of new probe (76%) as a dark blue solid. R_*f*_ = 0.25 (EtOAc/*n*-Hexane = 1/1 (v/v)).; Mp = 205 °C.; ^1^H NMR (300 MHz, *d*_6_-DMSO): δ = 8.10 (d, 2H, *J* = 9.63 Hz), 7.98 (d, 1H, *J* = 7.98 Hz), 7.56–7.41 (m, 5H), 7.04 (dd, 1H, *J* = 11.55 Hz, 14.85 Hz), 6.76 (d, 2H, *J* = 8.85 Hz), 4.79 (t, 1H, *J* = 5.30 Hz), 3.56 (t, 2H, *J* = 5.24 Hz), 3.49 (t, 2H, *J* = 5.29 Hz), 3.03 (s, 3H) ppm.; ^13^C NMR (75 MHz, *d*_6_-DMSO): δ = 163.7, 153.5, 151.8, 150.4, 149.1, 134.1, 131.0, 127.3, 126.1, 122.8, 122.5, 122.5, 118.0, 116.3, 112.2, 101.3, 58.5, 54.1 ppm.; IR (KBr): 3406, 2923, 2342, 2210, 1609, 1581, 1542, 1469, 1427, 1381, 1164 cm^–1^.; HRMS (ESI) *m*/*z* calcd. for C_21_H_20_N_3_OS ([M + H]^+^): 362.1327. Found 362.1324.

### Protein Expression and Purification

The constructs of the C-terminal domain of TDP-43 (residues 266-414) were prepared using a hexahistidine tag (His_6_ tag).^19^ The protein expression and purification are described in our previous publications.^21,27^ In short, the overexpressed protein was extracted from inclusion bodies using 8 M urea and purified using a nickel-charged immobilized metal-ion affinity chromatography column (Qiagen, Inc.) and then a C4 reverse phase column (Thermo Scientific, Inc.) using an HPLC system. The purified sample was lyophilized for storage. Additional treatments were applied in this study to control the sample quality and to maintain the consistency of kinetic aggregation assays. These precautions are critical to prevent the formation of preaggregates or “seeds” in the stored sample powder, which can result from residual moisture. Before each experiment, the dry protein powder was dissolved in 20 mM Tris with 6 M GdnHCl at pH 8.0. The protein solution was acidified to pH 2.0–3.0 with 50% trifluoroacetic acid and purified with a C4 reverse phase column using an HPLC system again. The eluted samples were lyophilized for 24 h. The protein powder was then incubated at 1 mg/mL concentration at room temperature for 24 h with 1,1,1,3,3,3-hexafluoro-2-propanol (HFIP) to disassemble possible aggregates and subsequently lyophilize for 24 h. The lyophilized samples were stored in a dry cabinet (relative humidity ∼35%) no more than a week before usage.

### Circular Dichroism Spectroscopy

Circular dichroism spectra were recorded using an AVIV model 410 spectropolarimeter with a 0.1 mm cuvette. Data were collected between 190 and 260 nm with an interval of 1 nm. Five measurements were coadded for each data point. All spectra were recorded at 303 K. All experiments were performed in triplicate. Samples (20 μM) were prepared in 20 mM sodium acetate buffer at pH 4.0, or 20 mM sodium phosphate buffer at pH 6.0.

### NMR Spectroscopy, Chemical Shift Assignment, and Data Analysis

^1^H–^15^N HSQC spectra were recorded using the standard pulse sequence with WATERGATE solvent suppression.^55,56^ Chemical shifts were assigned using standard HNCA, HN(CO)CA, HNCO, HN(CA)CO, CBCA(CO)NH, and HNCACB experiments acquired with nonuniform sampling (25%)^57,58^ at 283 K. All data were recorded using a Bruker AVIII 600 MHz spectrometer with a cryogenic probe.

The data were processed using NMRPipe.^59^ Chemical shifts were assigned using the automated assignment scheme^60^ implemented in NMRFAM-Sparky,^61^ and then confirmed manually. Secondary chemical shift analysis was performed using Kjaergaard et al.’s database of random-coil shifts.^62^ Secondary structure populations were estimated using δ2D.^63^ No chemical shifts were missing around the critical α-helical region, such that the secondary structure estimates for the constructs were made using the same number of chemical shifts.

### Aggregation Assay

ThT^ene–OH^ and ThT were prepared in pure dimethyl sulfoxide (DMSO) as stock solutions at a concentration of 10 mM. The stock solutions were diluted to the desired concentrations. The HFIP-treated and lyophilized protein powder was first dissolved in buffer, and the protein solution was centrifuged at 10,000*g* at room temperature for 3 min to remove potential aggregates. The concentration of the protein sample was determined by measuring the absorbance at 280 nm using a Nanodrop UV–visible spectrometer (Thermo Scientific, Inc.) with the appropriate extinction coefficient. The protein solution was diluted to the indicated concentrations with buffer solutions with the DMSO concentrations fixed at 1% (v/v). Mixtures containing protein and dye were loaded into a black-bottom 96-well polystyrene microplate (Greiner Bio-One International) with 100 μL for each well and then sealed with Mylar plate sealers (All Line Technology Co., Ltd.). Fluorescence reading was obtained using a SpectraMax M2 plate reader (Molecular devices) at 30 °C. The plates were shaken for 5 s before each read. For the long-period ThT assays (Figure 5), the plate was incubated at 30 °C without shaking between the daily measurements. Fluorescence intensity was recorded at λ_ex._/λ_em._ = 440 nm/480 nm for ThT and λ_ex._/λ_em._ = 510 nm/625 nm for ThT^ene–OH^. Each sample was measured in quadruplicate or quintuplicate. Tryptophan fluorescence spectra were recorded with λ_ex._ = 280 nm with temperature control at 25 °C for the solution and gelation samples.

### Preparation and Quantification of Hydrogels

The HFIP-treated and lyophilized protein powder was dissolved in the indicated conditions. Protein samples of 10 μL were aliquoted into 0.2 mL PCR tubes (Gunster Biotech Co., Ltd.) and then kept at 25 °C in an incubator over time. The levels of gelation were quantified by inserting a 3-μL capillary tube (ID0.0136 × OD0.0340/in.; Drummond Scientific Company) to the bottom of the PCR tube for 5 min, followed by measuring the height of the drawn-up sample in the tube.

### Small/Wide-Angle X-ray Scattering

The SAXS/WAXS data were recorded on the TPS 13A BioSAXS beamline at the National Synchrotron Radiation Research Center, Taiwan. Approximately 80 μL of protein solutions with a concentration of 10 mM were loaded into a 4-loading rocking cell (1.2–5 mm X-ray path length). The cell was then sealed to avoid evaporation. All the scattering profiles were corrected for the scatterings from air and an empty cell.

### Microscopic Analysis

Protein samples in the specified conditions were loaded into a quartz cuvette with a depth of 0.1 mm. Micrographs were collected using a Leica DM2500 microscope equipped with a Flexacam C5 camera.

### Negative-Stain Transmission Electron Microscopy

Lyophilized protein powder was dissolved in the specified buffer conditions and concentrations. The protein solution was then incubated at 30 °C for different periods to form fibrils. Following incubation, an aliquot of 5 μL of fibrillar sample solution was deposited onto 200-mesh Formvar carbon-coated copper grids. After allowing the sample to stand for 1 min, excess liquid was removed using filter paper. The grids were stained with 2% uranyl acetate aqueous solution for 1 min, followed by washes with double-distilled water. The grids were placed in a dry cabinet for over a week before TEM imaging. TEM images were acquired using a JEOL JEM-2000EXII microscope operating at 100 kV. Images were taken at magnifications ranging from 30,000× to 100,000×.

## Data Availability

All data and analyses collected in this study are deposited or available upon request.

## Supporting Information Available

Experimental procedures, characterizations, and analytical data for the synthesized compounds related to this study. It includes the synthesis protocols, methods for calculating quantum yield, and detailed descriptions of the physical properties and spectral data of the compounds involved (PDF)
