# Supplementary material for: TDP-43 Amyloid Fibril Formation via Phase Separation-Related and -Unrelated Pathways
Source: ACS Chem Neurosci. 2024 Oct 3;15(20):3767–75. doi: 10.1021/acschemneuro.4c00503 (PMC11488477; doi:10.1021/acschemneuro.4c00503)
Supplement: Supplementary file 1 — cn4c00503_si_001.pdf [file cn4c00503_si_001.pdf]

Supporting information

of

TDP-43 amyloid fibril formation via phase separation-related and -unrelated pathways

Pin-Han Lin[1], Guan-Wei Wu[1], Yu-Hao Lin[1], Jing-Rou Huang[1], U-Ser Jeng[4], Wei-Min Liu[5,*], and Jie-rong Huang[1,2,3,*]

[1]*Institute of Biochemistry and Molecular Biology, National Yang Ming Chiao Tung University, No. 155 Section 2, Li-nong Street, Taipei, Taiwan*
[2]*Institute of Biomedical Informatics, National Yang Ming Chiao Tung University, No. 155 Section 2, Li-nong Street, Taipei, Taiwan*
[3]*Department of Life Sciences and Institute of Genome Sciences, National Yang Ming Chiao Tung University, No. 155 Section 2, Li-nong Street, Taipei, Taiwan*
[4]*National Synchrotron Radiation Research Center, Hsinchu, Taiwan*
[5]*Department of Chemistry, Fu Jen Catholic University, No.510, Zhongzheng Rd., New Taipei City, Taiwan*

To whom correspondence should be addressed: jierongh@nycu.edu.tw, 133797@mail.fju.edu.tw

General remarks

^{1}H (300 MHz) and ^{13}C (75 MHz) NMR spectra were recorded with Bruker Avance III 300 MHz. NMR spectra were recorded in $CDCl_3$ or DMSO. Chloroform signals (δ = 7.26 ppm in ^{1}H NMR; δ = 77.16 ppm in ^{13}C NMR) and DMSO signals (δ = 2.50 ppm in ^{1}H NMR; δ = 39.52 ppm in ^{13}C NMR) were used as an internal standard. Splitting patterns are reported as follows: s (singlet), d (doublet), t (triplet), q (quartet), and m (multiplet). Coupling constants (*J*) are reported in Hz. IR spectra were recorded with a Bruker Tensor 27 Fourier-Transformed Infrared (FTIR) spectrometer or a PerkinElmer Spectrum Two FT-IR spectrometer, and data are reported in cm^{-1}. High-resolution mass spectrometry (HRMS) was carried out with a Thermo Finnigan LCQ Advantage (ESI-MS). UV/Vis absorptions and fluorescent emissions were carried out on a SpectraMax M2 microplate reader. Melting points were recorded with an MP-2D melting point instrument, and those were uncorrected. Reactions were monitored by *thin-layer chromatography* (*TLC*) on pre-coated sheets (Merck Art. 60 F_{254}, 0.25 mm) with detection by UV absorption (254 nm). Flash chromatography was performed on FUJI silysia chemistry MB-70-40/75. Yields of products refer to chromatographically purified or crystalline purified products unless otherwise stated. All reactions that required anhydrous conditions were carried out under N_2 or Ar. Dichloromethane (CH_2Cl_2) was dried with CaH_2 and then distilled. Tetrahydrofuran (THF) was dried overnight at 4Å molecular sieves (5 % w/v). Ethanol was dried overnight over 3 Å molecular sieves (5 % w/v).

Table S1.

$\lambda_{Abs.}$ (nm)^{a}	$\lambda_{Em.}$ (nm)^{a}	Quantum yield (%)	log P^{b}
480 nm	640 nm	2.42	4.64

^{a}Wavelengths of absorbance maxima and wavelengths of emission maxima of the modified probe were determined with 20 μM of the compounds in a 20 mM sodium phosphate buffer at pH 6.0. ^{b}Log P values were calculated by using online the ALOGPS 2.1 program.

Fluorescence measurement of the modified probe

Absorption and fluorescence emission wavelength were determined with 20 μM of the compound in a 20 mM sodium phosphate buffer at pH 6.0 by SpectraMax M2 multimode plate reader (Molecular Device, USA).

Calculation of Quantum yield

Methylene blue was used as a standard to determine the quantum yield of the modified probe. The quantum yield was calculated using the following equation,

$$\mathrm{QY} = QY_{ref} \frac{\eta^2}{{\eta_{ref}}^2} \frac{I}{A} \frac{A_{ref}}{I_{ref}}$$

where QY is the fluorescence quantum yield of the sample to be examined; QY_{ref} is the fluorescence quantum yield of the standard substance; η is the refractive index of the solvent for the sample to be tested; η_{ref} is the refractive index of the solvent for the standard; *I* is the integrated fluorescence intensity of the sample to be tested; I_{ref} is the integrated fluorescence intensity of the standard; *A* is the absorbance value at the excitation wavelength of the sample to be tested; A_{ref} is the absorbance value at the excitation wavelength of the standard. The quantum yield of methylene blue is 3% in methanol.[1]

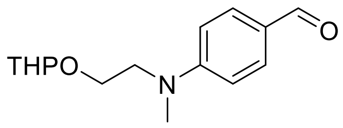

4-(methyl(2-((tetrahydro-2H-pyran-2-yl)oxy)ethyl)amino)benzaldehyde (SI)

To a solution of *N*-methyl-*N*-(2-hydroxyethyl)-4-aminobenzaldehyde (2 g, 11.16 mmole) and pyridinium *p*-toluenesulfonate (701 mg, 2.79 mmol) in anhydrous CH_2Cl_2 (40 mL) was added 3,4-dihydro-*2H*-pyran (3.1 mL, 33.48 mmol). The reaction mixture was stirred at room temperature for 37 h. The solvent was removed under vacuum, and the crude product was purified by silica gel chromatography to obtain 2.79 g (95 %) of compound **SI** as yellow oil. R_f = 0.30 (EtOAc/*n*-Hexane = 1/2 (v/v)).; ^{1}H NMR (300 MHz, $CDCl_3$): δ = 9.72 (s, 1H), 7.71 (d, 2H, *J* = 8.91 Hz), 6.74 (d, 2H, *J* = 8.94 Hz), 4.57 (t, 1H, *J* = 3.12 Hz), 3.96-3.89 (m, 1H), 3.80-3.72 (m, 1H), 3.70-3.56 (m, 3H), 3.49-3.42 (m, 1H), 3.10 (s, 3H), 1.80-1.62 (m, 3H), 1.59-1.46 (m, 4H) ppm.; ^{13}C NMR (75 MHz, $CDCl_3$): δ = 190.39, 153.76, 132.15, 125.37, 111.21, 99.14, 64.75, 62.29, 52.25, 39.31, 30.67, 25.46, 19.42 ppm. The ^{1}H and ^{13}C NMR spectra agreed with the reported literature.[2]

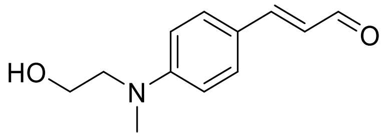

(*E*)-3-(4-((2-hydroxyethyl)(methyl)amino)phenyl)acrylaldehyde (SII)

A solution of (1,3-dioxolan-2-ylmethyl)triphenylphosphonium bromide (4.3 g, 12.4 mmol) in anhydrous THF (20 mL) was added with 60 % NaH in mineral oil (1.6 g, 39.7 mmol), and the solution mixture was stirred at room temperature. After stirring for 30 minutes, compound **SI** dissolved in anhydrous THF (15 mL) was added to the reaction mixture, and the reaction was heated to reflux for 6.5 h. The mixture was quenched with H_2O under an ice bath and extracted with EtOAc. The organic layer was dried over $MgSO_4$, and the solvent was removed under vacuum. The residue was purified by silica gel chromatography (EtOAc/*n*-Hexane = 1/4 (v/v)) to give a yellow oily liquid and was used for the next step. To a solution of the obtained oily liquid in THF (20 mL), 2M HCl $_{(aq.)}$ (20 mL) was added and the reaction mixture was stirred at room temperature for 20 h. The mixture was neutralized with 10 % NaOH $_{(aq.)}$ and was extracted with EtOAc. The organic layer was dried over $MgSO_4$, and the solvent was removed under vacuum. The crude product was further purified by silica gel chromatography to afford 779 mg (77 %, two steps) of compound **SII** as a yellow solid.

R_f = 0.15 (EtOAc/*n*-Hexane = 1/1 (v/v)).; Mp = 92 ˚C.; ^{1}H NMR (300 MHz, $CDCl_3$): δ = 9.54 (d, 1H, *J* = 7.92 Hz), 7.42 (d, 2H, *J* = 8.94 Hz), 7.34 (d, 1H, *J* = 15.66 Hz), 6.74 (d, 2H, *J* = 9.00 Hz), 6.49 (dd, 1H, J = 7.86 Hz, 15.57 Hz), 3.85 (t, 2H, *J* = 5.73 Hz), 3.58 (t, 2H, *J* = 5.67 Hz), 3.08 (s, 3H) ppm.; ^{13}C NMR (75 MHz, $CDCl_3$): δ = 194.05, 154.17, 151.99, 130.79, 123.78, 122.08, 112.01, 60.18, 54.55, 39.09 ppm. The ^{1}H and ^{13}C NMR spectra agreed with the reported literature.[2]

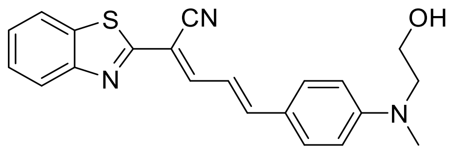

(2*E*,4*E*)-2-(benzo[d]thiazol-2-yl)-5-(4-((2-hydroxyethyl)(methyl)amino)phenyl)penta-2,4-dienenitrile (the modified probe)

To a solution of 2-benzothiazoleacetonitrile (39 mg, 0.22 mmol) and compound **SII** (46 mg, 0.22 mmol) in absolute ethanol (5 mL) was added three drops of piperidine. The resulting mixture was heated to reflux for 14.5 h. The reaction mixture was then cooled to room temperature and the solvent was removed under reduced pressure. The concentrated crude residue was recrystallized from CH_2Cl_2/MeOH co-solvent to give 61 mg of new probe (76%) as a dark blue solid. R_f = 0.25 (EtOAc/*n*-Hexane = 1/1 (v/v)).; Mp = 205 ˚C.; ^{1}H NMR (300 MHz, $CDCl_3$): δ = 8.10 (d, 2H, *J* = 9.63 Hz), 7.98 (d, 1H, *J* = 7.98 Hz), 7.56-7.41 (m, 5H), 7.04 (dd, 1H, *J* = 11.55 Hz, 14.85 Hz), 6.76 (d, 2H, *J* = 8.85 Hz), 4.79 (t, 1H, *J* = 5.30 Hz), 3.56 (t, 2H, *J* = 5.24 Hz), 3.49 (t, 2H, *J* = 5.29 Hz), 3.03 (s, 3H) ppm.; ^{13}C NMR (75 MHz, $CDCl_3$): δ = 163.72, 153.45, 151.82, 150.37, 149.07, 134.14, 131.01, 127.34, 126.06, 122.82, 122.52, 122.48, 118.03, 116.27, 112.22, 101.32, 58.52, 54.09 ppm.; IR (KBr): 3406, 2923, 2342, 2210, 1609, 1581, 1542, 1469, 1427, 1381, 1164 cm^{-1}.; HRMS (ESI) m/z calcd. for $C_{21}H_{20}N_3OS$ ($[M+H]^+$): 362.1327. Found 362.1324.

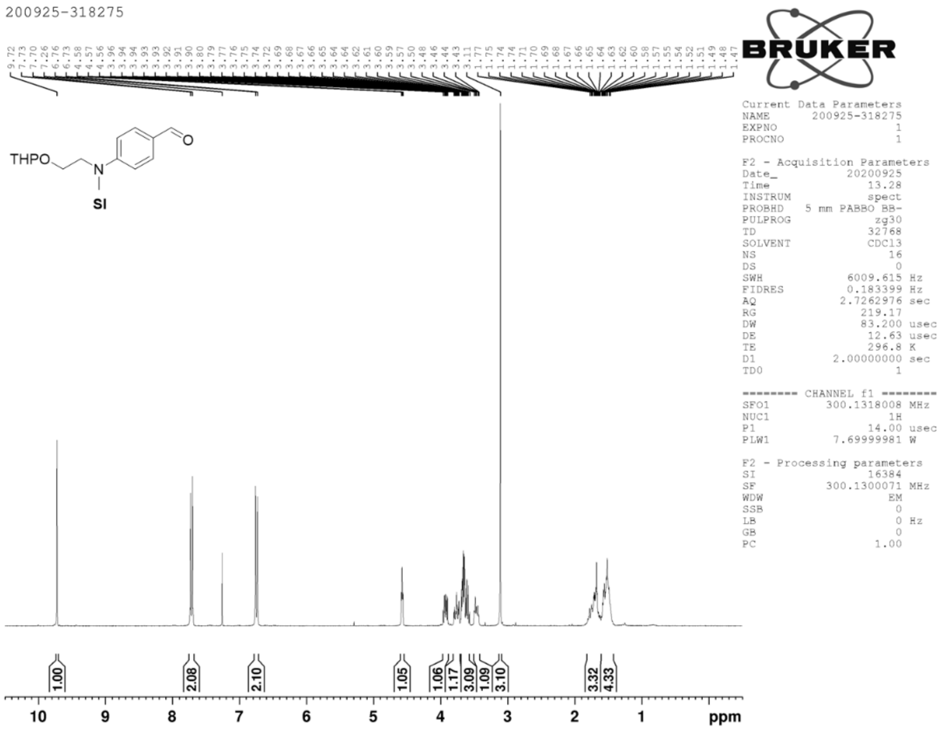

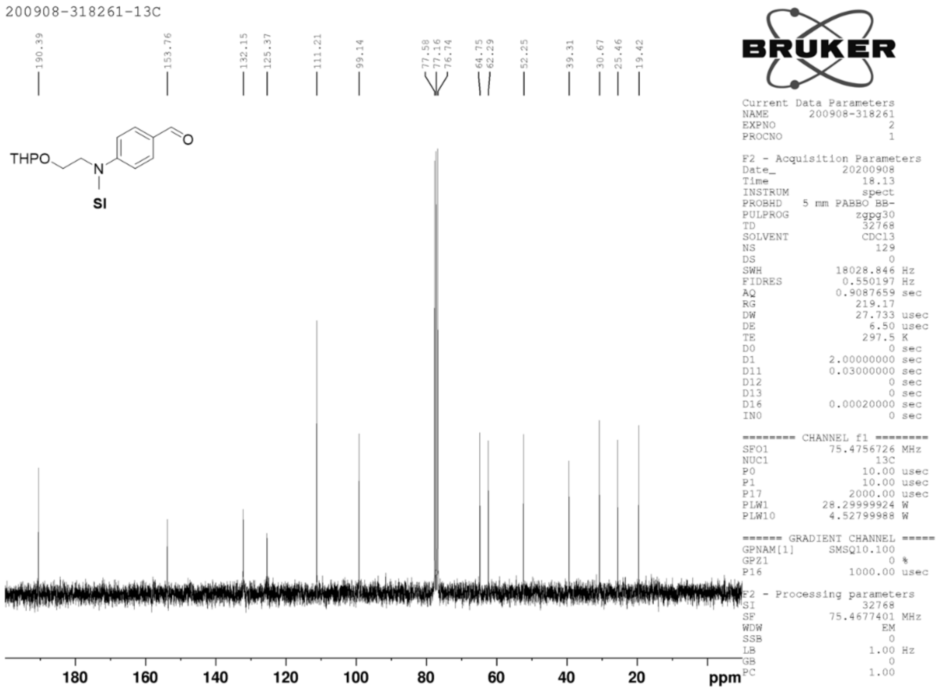

The ^{1}H and ^{13}C spectra of compound **SI**.

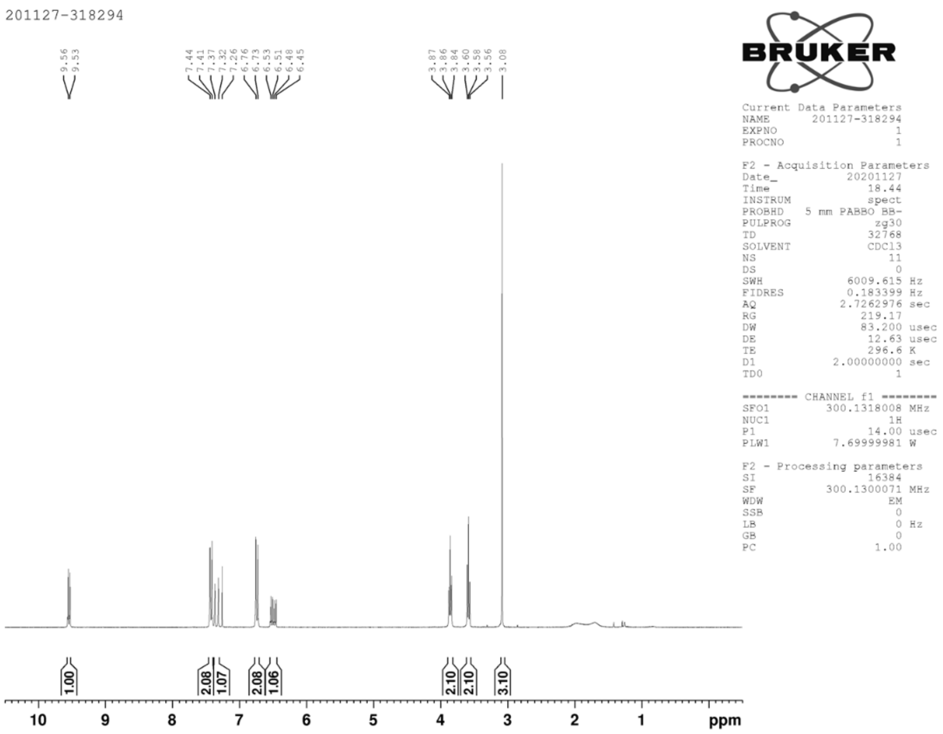

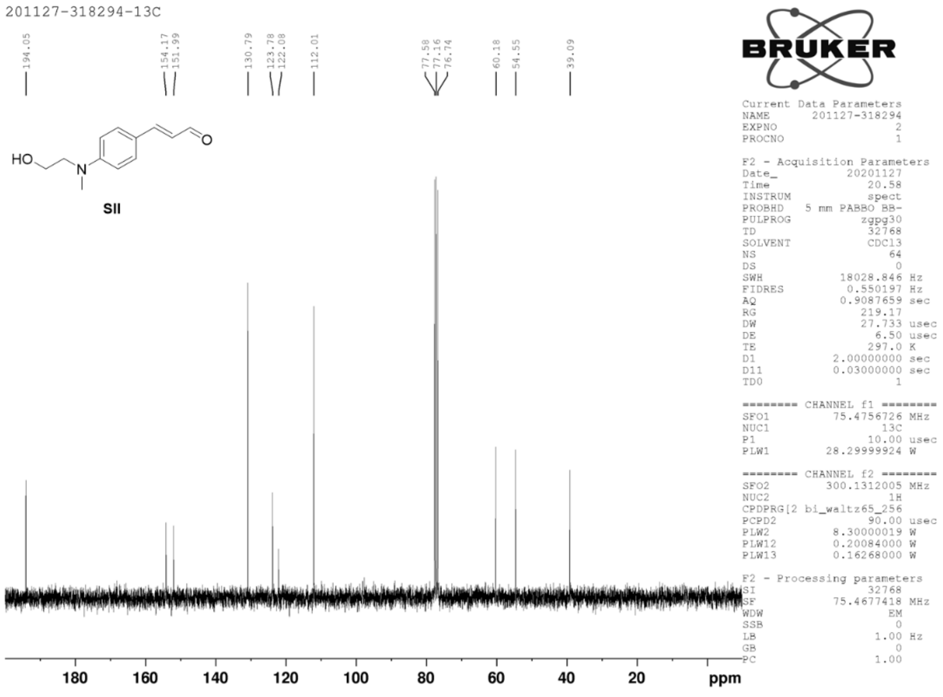

The ^{1}H and ^{13}C spectra of compound **SII**.

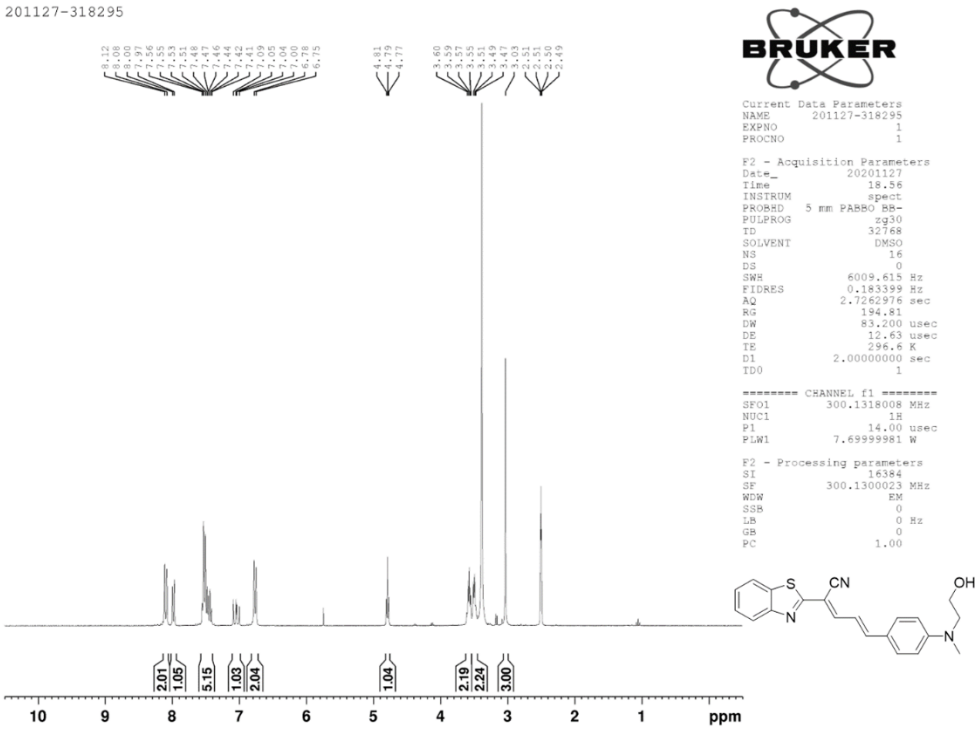

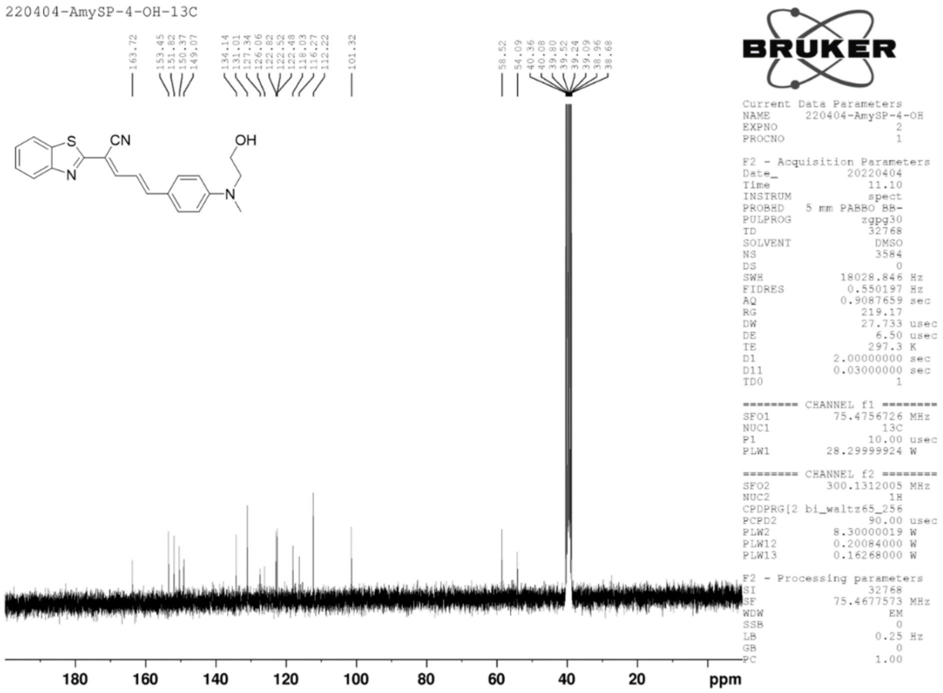

The ^{1}H and ^{13}C spectra of the new probe.

Elemental Composition Report Page 1

Single Mass Analysis
Tolerance = 500.0 PPM / DBE: min = -10.0, max = 100.0
Element prediction: Off
Number of isotope peaks used for i-FIT = 3

Monoisotopic Mass, Even Electron Ions
1556 formula(e) evaluated with 556 results within limits (up to 20 closest results for each mass)
Elements Used:
C: 1-100 H: 1-100 N: 1-10 O: 1-10 S: 1-3 I: 0-1

AmySP-4-OH
201217esl22 293 (2.866) Cm (293:296-(283:285+306:307))

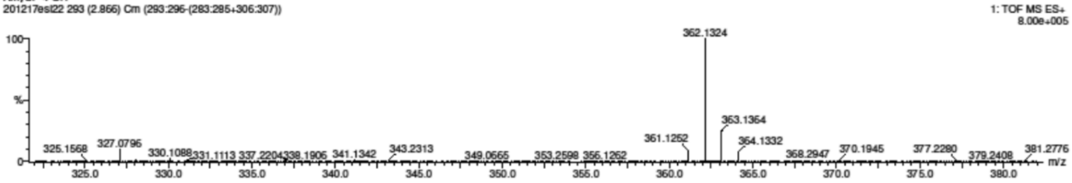

Minimum:				-10.0	
Maximum:		5.0	500.0	100.0	

Mass	Calc. Mass	mDa	PPM	DBE	Formula
362.1324	362.1327	-0.3	-0.8	13.5	C21 H20 N3 O S
	362.1321	0.3	0.8	4.5	C13 H24 N5 O3 S2
	362.1314	1.0	2.8	-4.5	C5 H28 N7 O5 S3
	362.1338	-1.4	-3.9	-6.5	C8 H33 N3 O2 S I
	362.1339	-1.5	-4.1	-8.5	C H28 N7 O10 S2
	362.1341	-1.7	-4.7	-5.5	C9 H32 N O7 S3
	362.1307	1.7	4.7	-0.5	C12 H28 N O7 S2
	362.1305	1.9	5.2	-3.5	C4 H24 N7 O10 S
	362.1346	-2.2	-6.1	0.5	C9 H24 N5 O8 S
	362.1301	2.3	6.4	-9.5	C4 H32 N3 O9 S3
	362.1354	-3.0	-8.3	-0.5	C10 H28 N5 O3 S3
	362.1359	-3.5	-9.7	5.5	C10 H20 N9 O4 S
	362.1361	-3.7	-10.2	8.5	C18 H24 N3 O S2
	362.1287	3.7	10.2	9.5	C16 H20 N5 O3 S
	362.1282	4.2	11.6	3.5	C16 H28 N O2 S3
	362.1280	4.4	12.2	0.5	C8 H24 N7 O5 S2
	362.1273	5.1	14.1	4.5	C15 H24 N O7 S
	362.1379	-5.5	-15.2	-4.5	C6 H28 N5 O8 S2
	362.1267	5.7	15.7	-4.5	C7 H28 N3 O9 S2
	362.1386	-6.2	-17.1	4.5	C14 H24 N3 O6 S

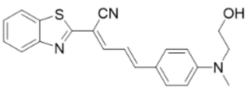

The HRMS spectrum of the modified probe.

Reference:

1. Olmsted, J., Calorimetric determinations of absolute fluorescence quantum yields. *The Journal of Physical Chemistry* **1979,** *83* (20), 2581-2584.

2. Watanabe, H.; Miki, Y.; Shimizu, Y.; Saji, H.; Ono, M., Synthesis and evaluation of novel two-photon fluorescence probes for in vivo imaging of amylin aggregates in the pancreas. *Dyes and Pigments* **2019,** *170*, 107615.
